# Role of the Frontal Cortex in Standing Postural Sway Tasks While Dual-Tasking: A Functional Near-Infrared Spectroscopy Study Examining Working Memory Capacity

**DOI:** 10.1155/2016/7053867

**Published:** 2016-02-03

**Authors:** Hiroyuki Fujita, Kenji Kasubuchi, Satoshi Wakata, Makoto Hiyamizu, Shu Morioka

**Affiliations:** ^1^Department of Neurorehabilitation, Graduate School of Health Sciences, Kio University, 4-2-2 Umami-naka, Koryo-cho, Kitakatsuragi-gun, Nara 635-0832, Japan; ^2^Department of Physical Therapy, Osaka Yukioka College of Health Science, Osaka, Japan; ^3^Neurorehabilitation Research Center, Kio University, Nara 635-0832, Japan

## Abstract

Posture control during a dual-task involves changing the distribution of attention resources between the cognitive and motor tasks and involves the frontal cortex working memory (WM). The present study aimed to better understand the impact of frontal lobe activity and WM capacity in postural control during a dual-task. High and low WM-span groups were compared using their reading span test scores. High and low WM capacity were compared based on cognitive and balance performance and hemoglobin oxygenation (oxyHb) levels during standing during single (S-S), standing during dual (S-D), one leg standing during single (O-S), and one leg standing during dual (O-D) tasks. For sway pass length, significant difference in only the O-D task was observed between both groups. oxyHb levels were markedly increased in the right dorsolateral prefrontal cortex and supplementary motor area in the high-span group during a dual-task. Therefore, WM capacity influenced the allocation of attentional resources and motor performance.

## 1. Introduction

Standing balance control is a complex sensorimotor action that is based on automated and reflexive spinal programs under the influence of several distinct and separate supraspinal centers in the brainstem, cerebellum, and cortex [1]. Several recent studies examining postural control have concluded that certain cognitive functions, such as attention, interact with motor function [2, 3]. A dual-task paradigm has often been used to elucidate the role of attention on postural control [4–6]. In recent years, there has been increasing research interest in the application of dual-task for rehabilitation. Woollacott and Shumway-Cook [7] reported that when participants were asked to perform a cognitive task at the same time as a postural control task, performance on both tasks decreased when the attentional resources needed exceeded participants' capacity. This supports a previous study that suggested that working memory (WM) capacity is limited [8]. Explanations generally revolve around the capacity-sharing theory, the bottleneck theory, or the multiple resource models theory. The capacity-sharing theory posits that attentional resources are limited in capacity. Thus, performance on two attention-demanding tasks causes deterioration in at least one of the tasks. When the time between the presentations of two or more stimuli is decreased, the processing time increases because of shared capacity limitations [9]. This theory assumes that it is possible to voluntarily allocate capacity to a specific task even when both tasks are overlearned and largely automatic. The bottleneck theory proposes that if two tasks are processed by the same neural processor or network, a bottleneck is created during information processing. The processing of the second task will be delayed until the processor has completed the first task. This theory explains delays in reaction times on the second task as a function of the temporal gap in the presentation of two stimuli. The multiple resource model suggests that processing may need several resources [10]. One of these theories claims that if two tasks do not share common resources, dual-task interference will not occur. For example, walking while performing a cognitive task might not cause any changes, but a second motor task that shares the same resources as walking will. Such studies have been interpreted to support all three models, and at present, there is no consensus as to the theory that best explains human information processing and dual-task costs.

When performing two simultaneous tasks, interference emerges. However, some conditions may allow for efficient distribution of attentional resources [11]. For example, Doumas et al. [2] created two conditions: an easy dual-task under stable conditions (e.g., a static standing position) and a difficult dual-task under an unstable condition (e.g., an unstable posture). The authors then assessed attentional resource allocation. In cases where a cognitive task was completed under a stable condition, elderly participants could allocate sufficient attentional resources to both the cognitive task and postural stabilization. However, when postural instability increased, attentional resources previously used for task performance tended to be allocated toward postural maintenance. Although a stable posture has been used to assess attentional resources allocated toward cognitive tasks, more stable attentional switching toward the appropriate challenge of maintaining a stable posture is required when allocating attentional resources. Attentional allocation is considered to be a key function of WM [12]. Regarding the relationship between WM and motor function, studies have suggested that the risk of falling increases when cognitive function is impaired due to advanced stage of Alzheimer's disease and other forms of dementia [13, 14]. Research has also found a significant correlation between task performance and walking speed in patients with mild cognitive impairment (MCI). Impaired motor performance is associated with decreased WM performance [15]. Schwenk et al. [16] studied the effect on dual-task performance in patients with dementia and suggested that specific rehabilitation program is effective in improving dual-task performance in patients with dementia. Verghese et al. [17] conducted a study to observe the effect of cognitive remediation on gait in sedentary seniors and suggested that cognition remediation improves mobility in older adults. Furthermore, decreased dual-task performance during motor task might result from these disorders. Reinforcing cognition to improve posture could be a focus of intervention and an important element in improving the quality of life in the elderly. Rehabilitation programs based on the improvement of cognitive capacity could be helpful for elderly persons who cannot undergo rehabilitation based on physical exercises. These programs could be offered in combination with or alternating with physical training that focuses on balance and strength. Therefore, decreased WM is suspected in patients who have difficulty completing motor tasks while engaging in another task. Thus, it is important to assess WM capacity in individuals during engagement in dual-tasks.

The reading span test (RST) is a widely used method for evaluating WM capacity [18]. Osaka et al. [19] used the RST to show that individuals with poor WM scores have difficulty changing the direction of their attention [20]. Furthermore, Osaka et al. [21] reported that the assessments of brain activity during an RST, similar to what was used in the present study, demonstrated that the dorsolateral prefrontal cortex (DLPFC) and anterior cingulate cortex (ACC) were more active in the high-span group than in the low-span group. These individuals struggle to suppress their focus on a particular target once attention has been deployed and is subsequently needed elsewhere. Thus, this research suggests that performance on primary and secondary tasks depends on an individual's WM capacity. However, variability in attentional function, which consists of the inhibition and allocation of attentional resources, due to the differences in WM capacity based on RST results might not be limited to the language domain. Engle et al. [22] determined that the relationship between attentional control functions and WM capacity is significant regardless of the cognitive domain. For instance, Conway et al. [23] reported that attentional control is not limited to the cognitive control and language processing. It is conceivable that WM capacity, as assessed by the RST, could be related to motor control ability. Those who score low on the RST and have difficulties with motor tasks probably struggle with allocating resources between simultaneous cognitive and motor inputs. It is predicted that a low scoring RST group has low DLPFC activity during motor function with dual-task.

In contrast, balance control is associated with attention, particularly during a dual-task [24]. Balance control is often associated with the DLPFC and supplemental motor area (SMA) [24, 25]. Activation in the prefrontal cortex may reflect attentional processing during the postural control because accumulated evidence suggests that the attentional ability plays an important role in postural control [11]. It has been reported that the frontal lobes play an important role in the allocation of attention. The DLPFC, in particular, plays a top-down supportive role for determining appropriate behavior and is considered to be important for the maintenance of attention when performing a task and attentional control comprises 2 main components, namely, conflict detection during task performance and allocation of attention to adjust the attentional resources when necessary [26]. In these subcomponents of the attentional process, the ability to allocate attention has been emphasized in the maintenance of postural stability [27]. Together with the assumption that the DLPFC is involved in the selective allocation of attention [28], these reports suggest that DLPFC activation is relevant to the attentional process for balance control [29].

It remains unclear how WM capacity, assessed with the RST, influences postural control during dual-task performance. Because it is assumed that attentional resources influence postural control, WM, due to its role in attentional allocation, is probably involved in postural control during task performance. Nevertheless, previous research has not yet assessed how attentional capacity relates to motor control. Therefore, the main aim of the present study was to examine how WM capacity, assessed with the RST, influences postural control and frontal lobe activity during a dual-task. We hypothesized that frontal lobe activation would be higher during standing with a complex condition compared with simple conditions. In addition, we further hypothesized that the high-capacity group, assessed with the RST, would demonstrate higher brain activity than the low-capacity group in complex conditions.

## 2. Methods

### 2.1. Participants

**Figure 1:**
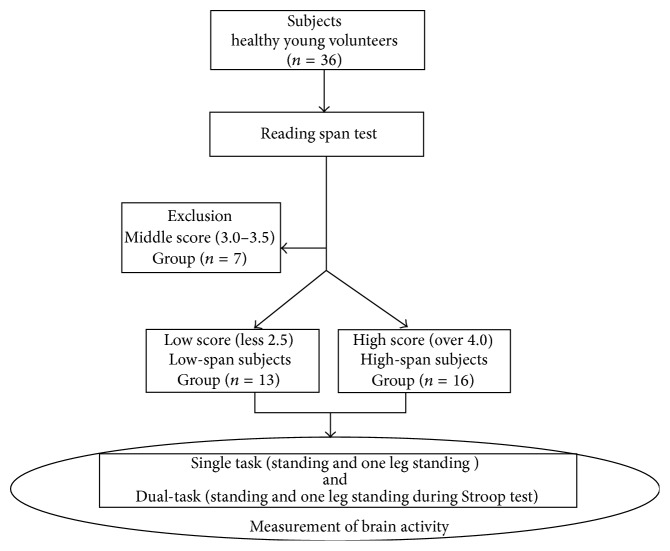
Flow diagram of study participants and allocation to group. Subjects were 36 healthy young volunteers who were divided into two groups based on their scores in the reading span test, Japanese edition: the first and second groups comprised subjects scoring high scores and a low score, respectively, and the average scorers were excluded. Postural sway was measured for 20 s, and the participants were requested to maintain a standing position while performing the Stroop test and the error number of the results was detected and assumed as an index. We measured (1) S-S: standing with single task, (2) S-D: standing with dual-task, (3) O-S: one leg standing with single task, and (4) O-D: one leg standing with dual-task. In addition, brain activity was measured when the participants were in these postures by fNIRS.

A total of 36 individuals (aged 20–29 years) with no history of cerebrovascular accidents, orthopedic disorders, or visual impairments were recruited. In addition, alcohol ingestion was forbidden before the experiment. To measure WM span, we administered the RST to all participants. In line with previous studies [21], we used the results to divide the participants into high and low WM-span groups: individuals scoring ≥4.0 were placed in the high-span group, whereas those scoring ≤2.5 were placed in the low-span group. Participants who scored 3.0 or 3.5 on the RST were excluded from the study. The study ultimately comprised 29 participants, with 16 and 13 in the high- and low-span groups, respectively. The demographic characteristics of the participants are summarized in Table 1, and a flow chart of the study is presented in Figure 1. All participants provided written informed consent prior to participating in the study, and the Kio University ethics review board approved its design.

### 2.2. Reading Span Test

The RST [18] is a widely known measure of WM span and is believed to be a useful indicator of cognitive ability. In the present study, we used the Japanese version of the RST developed by Osaka et al. [19]. First, in the one-sentence task, the participants were shown five slides in succession with one sentence on each slide. Each sentence was different, but the difficulty level of the words was the same. If the one-sentence task was successfully completed, the test progressed to the two-sentence task in which each slide contained 2 sentences with a total of 5 slides shown in succession. This procedure was repeated in the subsequent three-, four-, and five-sentence tasks, with five slides shown in each task. Each task sentence was no more than one line in length. The sentences were displayed in black text on a white background on a 15 × 21 cm computer screen. Participants were instructed to read each sentence aloud within 5 s of seeing the slide while simultaneously memorizing the underlined target words in each sentence. The participants were then asked to recall the target words. The target words could be reproduced in any order; however, to avoid a recency effect, participants were forbidden from reproducing the target word from the last sentence first. Participants who correctly answered three of the five problems passed that level of the test and progressed to the next task level. Results were scored according to the most difficult task passed, with the number of points given equal to the number of sentences in the task. For example, the participants who passed the two-sentence task but did not reach a higher level received a score of two. Therefore, the score corresponded to the highest level achieved. The participants who answered two out of five problems correctly were given additional 0.5 points. The sentences presented and the time allotted for the presentation and reproduction were all in accordance with the methods used in previous studies [21]. No target words within a task were definitionally related. Based on these scores, participants were divided into a high WM-span group and a low WM-span group.

### 2.3. Standing Postural Control

We assessed the participants' ability to maintain a standing posture during the execution of single and dual-tasks. Center of pressure displacement was recorded at a sampling frequency of 50 Hz, as recommended by Ruhe et al. [30], using a pressure distribution measuring device (Puredasu MD-1000; Anima Corp., Tokyo) for assessing the sway path length. Participants maintained a standing posture for 20 s during the execution of single and dual-tasks. Participants focused on a target word, whose meaning and color were the same, on a screen while standing still (standing during a single task: S-S task) and were then asked to stand still on one leg (one leg standing during a single task: O-S task). These two conditions (S-S and O-S tasks) were considered as a single task. Subsequently, participants were assigned the dual-task of standing (standing during a dual-task: S-D task) and standing still on one leg (one leg standing during a dual-task: O-D task) for 20 s while performing a Stroop test. Participants were asked to minimize their physical sway as much as possible and to concentrate on the cognitive task while maintaining a standing posture. Participants stood on the force platform during the Stroop test and were instructed to counterbalance by explicitly minimizing the postural changes. In all conditions, participants were asked to avoid large movements, keep their arms at their sides, and look straight ahead at the computer monitor used for the cognitive task.

### 2.4. Stroop Test

The Stroop test is used to assess an individual's impaired response inhibition due to frontal lobe dysfunction, problems with simultaneous interference, and ability to divide attention [31]. Although various secondary tasks are used as part of a dual-task methodology, the Stroop test differs from calculations or letter repetition, in that, there is no difference in difficulty among the stimuli. We chose the Stroop test because the effects attributable to differences in learning ability among participants are minimal. Participants were shown 20 cards, which contained the names of colors written in different colors on a screen for 1 s each and asked to state the color in which the word was written. The number of incorrect responses was subtracted from 20 to calculate the number of correct responses. To reduce the effects of fatigue and Stroop test experience, subjects took 2 min breaks between tests. Stroop tests were also administered to the subjects while sitting to assess their cognitive ability and confirm that they did not have any problems with cognitive function. To prevent both physical fatigue due to performing the tasks in proper chronological order and cognitive task learning effects that would influence outcomes, the tasks were randomized for each subject using a table of random numbers (Microsoft Office Excel 2007, Microsoft Co., Tokyo, Japan).

### 2.5. Functional Near-Infrared Spectroscopy

**Figure 2:**
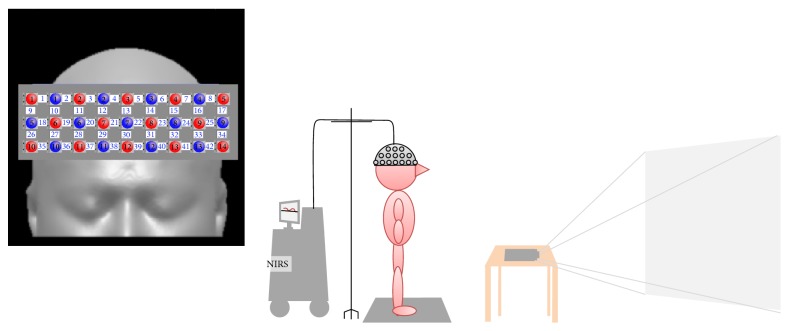
fNIRS measurement channels and task setting. Supplementary motor area: channels 13, 21, 22, and 30. Premotor cortex: channels 11, 12, 20, 28, and 29 (right) and 14, 15, 23, 31, and 32 (left). Dorsolateral prefrontal cortex: channels 18, 26, 27, and 35 (right) and 25, 33, 34, and 42 (left). Cz: T3. Protocol Rest 10 s-task 20 s-rest 10 s, 3 set × 4 tasks. The subject was given 4 random tasks and a sufficient break between the tasks.

Cortical activation was assessed by task-related changes in hemoglobin oxygenation using an fNIRS system (FOIRE-3000; SHIMADZU Corp., Kyoto, Japan). The system comprised 13 near-infrared light (780, 805, and 830 nm) source fibers and 14 detectors, thus comprising 42 source-detector pairs (channels 1–42). The system detected changes in the cortical concentration (mM·cm) of oxyHb, deoxyHb, and total hemoglobin by applying a modified Beer-Lambert law to data acquired simultaneously at a sampling rate of 190 ms [32]. Sampling frequency was 10 Hz, the processed moving average was 5 s, a 0.5 Hz low-pass filter was used to remove the effects of Mayer waves, and a 0.01 Hz high-pass filter was used to remove baseline drift. The task was repeated three times and the data from each of the three task blocks were averaged. The vertex (Cz) position of the international 10–20 system was used to ensure consistent optode placement. The interoptode distance was 3 cm. The fNIRS topographic map covered the frontal area. The probe locations were measured using a 3D position measuring system (FASTRAC; Polhemus, Colchester, VT, USA), and stochastic registration of the Montreal Neurological Institute (MNI) brain coordinates was performed using NIRS-statistical parametric mapping [33]. The image expressed these threshold positions on MNI, the Talairach standard brain coordinate axis, and expressed it as a probability distribution. Based on these data, a reference brain database was built [34]. The brain locations corresponding to each channel were identified, and the area was divided into five regions of interest (ROIs; Figure 2) based on the functional anatomy of the supplementary motor area (SMA), premotor cortex (PMC), and dorsolateral prefrontal cortex (DLPFC). The SMA was covered by channels 13, 21, 22, and 30; the left and right PMC by channels 14, 15, 23, 31, and 32 and channels 11, 12, 20, 28, and 29, respectively; and the left and right DLPFC by channels 25, 33, 34, and 42 and channels 18, 26, 27, and 35, respectively.

Cortical activation was assessed as task-related increases in oxyHb levels because oxyHb is much more sensitive to task-related changes than deoxyHb [35]. The oxyHb data from each channel of each subject were normalized by linear transformation, so that the mean ± standard deviation (SD) of the oxyHb levels in the initial 10 s of the rest period was 0 ± 1 effect size (ES). This normalization was also useful for circumventing the influence of differential path length factors among the subjects and that of the cortical regions on oxyHb levels [36, 37]. ES  (*d*) were calculated according to Schroeter et al. [38] as the difference of the means of task and rest conditions divided by the standard deviation of the rest condition:

(1) $$\begin{array}{c} d=\frac{\left(m_{1}-m_{2}\right)}{s}. \end{array}$$

Accordingly, *m*_1_ and *m*_2_ are the mean signal strengths during the task (*m*_1_) and rest (*m*_2_) conditions and *s* is the standard deviation of the rest condition. Comparisons between groups were calculated during the S-S, S-D, O-S, and O-D tasks. Regional changes in oxyHb levels were obtained from each channel in each subject. The data were averaged in each channel and then the values for each ROI were obtained by averaging the data from the relevant channels in each subject. Comparisons of regional activation during tasks were calculated as the mean oxyHb value in the task minus the mean oxyHb value in the rest phase.

### 2.6. Data Analysis

Stroop test scores for each postural position were compared using the Friedman test. The sway path length between each condition of the high- and low-span groups was compared using two-way repeated measures ANOVAs with group (high, low) as a between-subjects factor and condition (S-S task, S-D task, O-S task, and O-D task) as a within-subjects factor. Moreover, to compare the activation in each region related to WM capacity, the ES was calculated for each task in each ROI. The ES in each ROI was then compared between the high- and low-span groups in each task using two-way repeated measures ANOVAs with group (high, low) as a between-subjects factor and condition (S-S task, S-D task, O-S task, and O-D task) as a within-subjects factor. To examine the relationship between central executive and standing postural sway task, the correlation between RST and the sway path length was analyzed using a spearman correlation coefficient. The level of statistical significance was set at *p* < 0.05.

## 3. Results

### 3.1. Cognitive and Balance Performance (Table 2)

In the Stroop test, there was no significant difference in the sitting, S-D, and O-D tasks between both groups. A two-way repeated measures ANOVA (see Table 2) revealed significant differences between postural conditions within both groups [*F*(3, 78) = 29.3, *p* < 0.05]. Furthermore, we observed an interaction between the groups with regard to postural condition [*F*(3, 78) = 3.15, *p* < 0.05] (Table 2). After applying a Bonferroni correction to these results, the only statistically significant difference observed between the high-span groups occurred in the sway path length during the S-D (*p* < 0.05). There was no significant difference in the other tasks (S-S task, S-D task, and O-S task) (*p* < 0.05) between the groups.

There was a significant negative correlation between the sway path length during the O-D task and RST (*r* = −0.44, *p* < 0.05). The sway path length observed during the S-S task as well as during the O-D and O-S tasks did not significantly correlate with RST (S-S task: *r* = −0.1, *p* = 0.5, S-D task: *r* = −0.002, *p* = 0.9, and O-S task: *r* = 0.07, *p* = 0.7).

### 3.2. Cortical Activation during the Four Tasks (Table 2)

A two-way repeated measures ANOVA (see Table 2) revealed significant differences in SMA between the high- and low-span groups [*F*(1,27) = 4.63, *p* < 0.05]. Statistically significant differences were also observed between postural conditions within both groups [*F*(3, 81) = 5.9, *p* < 0.05]. Furthermore, we observed an interaction between the groups with regard to postural condition [*F*(3, 81) = 4.55, *p* < 0.05] (see Table 2). After applying a Bonferroni correction to these results, the statistically significant difference was observed between the high- and low-span groups in brain activity during the O-D task [*F*(1, 103) = 15.7, *p* < 0.05] (see Table 2). Significant effects were observed in intergroup and intragroup comparisons between the high- and low-span groups according to postural condition. Intragroup comparisons revealed that SMA was significantly greater during the O-S and O-D tasks in the high-span groups compared with that during the S-S task. A two-way repeated measures ANOVA (see Table 2) revealed no significant differences in the DLPFC (right) between the high- and low-span groups [*F*(1, 27) = 1.11, *p* > 0.05]. Statistically significant differences were observed between postural conditions within both groups [*F*(3, 81) = 3.24, *p* < 0.05]. Furthermore, there were no significant interactions between the groups with regard to postural condition [*F*(3, 81) = 2.09, *p* < 0.05] (see Table 2). After applying a Bonferroni correction to these results, the DLPFC (right) activation was significantly greater during the O-S (*p* < 0.05) and O-D tasks (*p* < 0.05) in the high-span groups compared with the S-S task. In addition, results of the present study showed that there were no significant differences in DLPFC (left), PFC (right), and PFC (left) activation between the groups and various postural conditions between the high- and low-span groups.

## 4. Discussion

There was no significant difference in the results of the Stroop test between the posture conditions for the two groups. Sway path length, a measure of postural control, was longer in the low-span group than in the high-span group during the O-D task.

There was no marked change in the sway path length or changes in the Stroop test among the high-span group, suggesting that participants in this group were able to allocate attentional resources to both postural maintenance and the Stroop test. Meanwhile, no deterioration in the Stroop performance in either posture was observed among the low-span group, but there was a significant difference in sway length during the O-D task when compared with the high-span group. Compared with the S-S task where posture was stable, greater attentional resources were required during the O-D task (where posture was unstable). Greater attention is required during the unstable posture conditions (difficult task condition) than during the stable posture conditions (easy task condition) [2, 3]. These results suggest that greater attentional resources and their appropriate allocation are required for postural stabilization during a dual-task; thus sway path length increased with increasing postural demands. Among the low-span group, however, a larger proportion of attention was allocated to the performance of the Stroop test and the allocation of attention to postural control was reduced. This resulted in insufficient attention being allocated to task achievement and reduced stability during standing. The young and healthy present study participants required few attentional resources to perform the Stroop test while maintaining a standing posture, with participants easily completing the task without experiencing dual-task interference. Regarding task precedence in a dual-task paradigm, Lajoie et al. [39] and Shumway-Cook and Woollacott [40] reported that elderly participants exhibited a posture-first strategy, prioritizing attentional resource allocation to posture maintenance (i.e., a motor task) above cognitive task performance. Doumas et al. [41] also reported that elderly participants performing a dual-task focused their attention on postural maintenance, whereas younger participants focused on performing the cognitive task. The present study incorporated a prolonged sway path length, and no impairment of Stroop test performance was observed in the low RST group. This finding suggests that younger individuals prioritized the Stroop test during the S-D task and O-D task. In addition, a significant negative correlation was observed between the sway path length during the O-D task and RST. These results indicate that postural sway is influenced not only by the demands of posture conditions during a dual-task but also by individual WM capacity. Specifically, when the low-span group allocated attention toward secondary task performance during the O-D condition, necessary attentional resources could not be allocated toward postural stabilization maintenance, and sway path length increased. The most common explanations for dual-task effects in posture-cognition studies have adopted a resource competition framework [7]. This framework appears well suited to studies with the elderly and patient populations. This is because the most commonly observed dual-task effect among these participants is an increase in measures of postural instability or a decline in cognitive task performance, both of which are interpreted as resulting from insufficient resources available for both tasks. Results from the present study were consistent with these findings, in that, focusing attention on one task reduced performance on the other [6]. For the high RST group, during the O-D task, attentional resources were properly suppressed for the Stroop test, ensuring sufficient resources would be available for postural control in the present study. There is a need for appropriate attentional allocation during execution of complex movement (O-D task). In addition, suitable division of attentional allocation is required to appropriately process information presented during an unstable situation. WM capacity limitations among the low-span group left those individuals susceptible to interference more so than the high-span group. Compared with the S-S task, where posture was stable, more attentional resources were required during the O-D task, where posture was unstable. It was verified that more attention was required during the unstable posture condition (difficult task condition) than during the stable posture condition (easy task condition) [3, 41]. These results suggest that greater attentional resources and their appropriate allocation were required for postural stabilization during the O-D task, where sway path length was prolonged compared with a stable standing posture.

Furthermore, the effect of these task conditions on frontal lobe activation was examined using fNIRS. The results demonstrated a significant increase in oxyHb concentration in the SMA and right DLPFC of the high-span group compared with those of the low-span group in the O-D task. There was no significant difference in any other cortical area between the high- and low-span groups in the O-D task. Moreover, for the other three conditions, there was no significant difference in any cortical area between the high- and low-span groups.

Significant activity of the SMA was observed in the O-D task in the high-span group. Several studies have demonstrated plasticity in the cortical and subcortical areas during the acquisition of new motor skills [42]. According to these studies, the SMA displayed increased activation from the early learning phase to the automatization phase. Furthermore, the SMA is activated during movement planning or when a previously learned sequence is executed. The SMA may have an important role in the learning phase of motor representation and planning [43] and in balance control and recovery [29]. In contrast, the SMA did not have a significant role in O-S task performance, if standing on one leg is considered to be a novel learning experience. Callicott et al. [44] reported that an excessive WM load resulted in a reduction in task performance and lesser activation of the frontal association area. Carlson et al. [45] reported that the frontal lobe was not appreciably activated by a 1-back task, which is widely regarded as the least difficult *n*-back task, which is a type of WM task. Therefore, there may be little activation of the frontal association area in response to a small or a large load, but a significant amount of activation may occur with moderate loads. Based on these studies, the exercise of standing on one foot may have been an easy task for healthy adults and therefore may not have triggered increased brain activity in response to this O-S task. In addition, standing on one leg is a more novel task than maintaining a standing posture with both feet. The implementation of this unfamiliar movement was presumably recognized as a new learning task, causing SMA activity to be observed while the O-D task was performed. Previous studies have shown that the SMA is involved in interlimb coordination [46] and postural control [47] in healthy participants. Increased activation of the SMA has also been observed during lower-limb movement coupled with gait recovery after stroke [48]. In that study, SMA activity was mainly observed during poor O-D task performance and mainly in the high-span group. The activity that was more meaningful than S-S task was accepted only in the high group, which was able to apply attention resource to a posture task appropriately. SMA activity was observed during O-D task performance by the high-span group. This O-D task of standing on one leg demands a great deal of ankle activity. The high group was able to focus their attention resource on performing a cognitive task and a posture task as needed. In this experiment, the high group was better able to minimize activity-associated agitation than the low group. Thus, enhanced SMA activation may reflect preparation for ankle joint movement in order to prevent body sway. In addition, the DLPFC plays an important role in WM by efficiently allocating attentional resources when individuals are performing simultaneous tasks [10, 20]. This area helps maintain attention and in particular spatial attention, which may be important for postural control [49]. Wallis and Miller [49] reported that the DLPFC plays an important role in physical activity performance by integrating external information with information on body positioning during the activity. Maki and McIlroy [24] examined the role of the prefrontal area in balance control and suggested that the participation of the prefrontal area relates to a suitable distribution of spatial attention. That is, the DLPFC may have an important role in attitude control during a dual-task. In the present study, a difference in right but not left DLPFC activation was observed between the high- and the low-span groups. Several studies have reported lateralization of postural control, with the right hemisphere having a greater role than the left hemisphere in postural control [50, 51]. Ugur et al. [50] reported that individuals with a right hemispheric lesion were more likely to fall after a stroke. They also reported that abnormalities in postural control or postural disorders were more frequently observed in this population, with the extreme aligning with the “Pusher Syndrome” [51]. Based on this evidence, it is believed that the right hemisphere plays a dominant role in postural control. Therefore, the present study's findings that differences in activation were observed in the right but not the left DLPFC are consistent with those of previous studies.

The observed differences in brain activity between the high- and low-span groups may be due to different levels of WM performance. In the low-span group, the increase of oxyHb concentration in the DLPFC and SMA observed in the high-span group may have been difficult to detect because of their low WM capacity and the high difficulty of the O-D task. Osaka et al. [21] reported that brain activity at the time of the RST (similar to that used in the present study) demonstrated that the DLPFC and ACC were more active in the high-span group than in the low-span group. Thus, it is possible that the low-span group in the present study was engaging the DLPFC and ACC less during complex tasks. Compared with maintaining a normal standing posture, the O-D requires more active integration of external information to maintain posture control during a cognitive task. Therefore, only subjects in the high-span group were able to properly allocate resources to the cognitive and motor tasks to complete the task. These results suggest that WM played an important role in allocating and changing the direction of attentional resources and that differing WM capacities may result in different brain activation patterns that may be influential in motor performance.

These study results have important implications for fall prevention. Specifically, to maintain balance during dual-task situations, an individual must be flexible to allocate their attention to balance control to prevent falls. Dual-task situations require the ability to allocate information-processing resources between the two relevant tasks while maintaining an adequate attentional set. Further studies should be conducted to determine whether the ability of elderly individuals to allocate attention contributes to their risk of falling. The limitations of this study included the fact that the RST was a complex task requiring multiple psychological components. The RST requires subjects to memorize the target word (storage), read the stimulus statement (processing), and conduct these tasks in parallel (control). It is also necessary to study what role the WM plays in posture control. Stroop task may be very simple for young people. Therefore, it might have been necessary to measure the reaction time along with counting the correct number. Furthermore, the Stroop test was employed as a secondary task for which a verbal response was prepared during a period of postural control. This may have induced changes regarding dynamic fluctuations in postural control, resulting in an increase in sway path length. Thus, reaction time of the Stroop test will be measured in future studies, and we need to consider the difficulty of the secondary task. Finally, our results may not be appropriate for generalization because the sample size was small in this study.

## 5. Conclusion

Our results suggest that differences in WM capacity affect not only the performance of physical activity but also brain activity. This study revealed that the WM capacity is involved in postural control. When evaluating balance and behavioral level when applying a dual-task in rehabilitation, there is also a need to evaluate WM as a cognitive function.

## Figures and Tables

**Table 1:** Profile of subjects.

	Subjects (*n* = 29)	High-span group (*n* = 16)	Low-span group (*n* = 13)	*p* value
Age (mean ± SD)^*∗*^	23.1 ± 2.6	22.5 ± 3.6	24.0 ± 3.1	*p* > 0.05
Height (mean ± SD)^*∗*^	164.9 ± 7.7	166.4 ± 9.4	164.9 ± 7.6	*p* > 0.05
Weight (mean ± SD)^*∗*^	58.7 ± 9.1	60.4 ± 10.3	58.8 ± 9.1	*p* > 0.05
Gender (male/female)^*∗∗*^	11/18	6/10	5/8	*p* > 0.05

^*∗*^ *t*-test; ^*∗∗*^chi-squared test.

**Table 2:** Comparison of brain activity and motor and cognitive function in high-span group and low-span group.

	Low-span group				High-span group			
	S-S task	O-S task	S-D task	O-D task	S-S task	O-S task	S-D task	O-D task
SMA^a,b,c^	0.06 ± 0.57	0.06 ± 0.67	0.22 ± 0.41	0.09 ± 0.45	−0.06 ± 0.31	0.55 ± 0.84^*∗*^	0.08 ± 0.52	0.96 ± 0.66^†*∗*^
PMC (right)	−0.01 ± 0.51	0.06 ± 0.71	0.18 ± 0.41	0.15 ± 0.81	0.03 ± 0.33	0.05 ± 0.94	0.38 ± 1.75	0.34 ± 0.85
PMC (left)	−0.04 ± 0.57	0.37 ± 2.03	0.07 ± 0.55	0.20 ± 0.76	−0.08 ± 0.42	0.35 ± 1.18	−0.08 ± 0.41	0.35 ± 1.17
DLPFC (right)^c^	0.11 ± 1.23	0.53 ± 0.81	0.55 ± 1.44	0.60 ± 1.29	0.31 ± 1.58	0.23 ± 1.70	0.35 ± 1.21	2.00 ± 1.78^†^
DLPFC (left)	−0.11 ± 0.58	0.39 ± 0.64	0.37 ± 1.16	0.24 ± 0.75	0.13 ± 1.51	0.76 ± 1.88	0.39 ± 0.94	1.17 ± 1.41
Sway path length (cm)^b,c^	68.3 ± 12.6	74.2 ± 14.0^†^	72.0 ± 10.8	94.0 ± 14.0^†^	71.3 ± 9.8	72.5 ± 9.7	74.9 ± 14.1	83.9 ± 7.60^†*∗*^

Two-way repeated measures analysis of variance								
Stroop test (number of correct answers)	19.8 ± 0.44 (sitting)		19.8 ± 0.4	19.6 ± 0.71	19.9 ± 0.27 (sitting)		19.8 ± 0.37	19.7 ± 0.63

S-S task: standing during a single task, O-S task: one leg standing during a single task, S-D task: standing during a dual-task, and O-D task: one leg standing during a dual-task.

(ES: mean ± S.D.)

^a^Significant main effect of group (*p* < 0.05), ^b^significant different group interaction (*p* < 0.01), ^c^significant main effect of condition (*p* < 0.05),^*∗*^significant different low-span group (*p* < 0.05), and ^†^significant different S-S task.
